# Sleep deprivation-induced memory impairment: exploring potential interventions

**DOI:** 10.3389/fpsyt.2024.1470976

**Published:** 2024-10-07

**Authors:** Yisheng Fan, Jianfeng Li, Shanfeng Qiao

**Affiliations:** ^1^ Department of Urology, Shuyang Hospital of Traditional Chinese Medicine, Jiangsu, China; ^2^ Department of Surgery, Yizheng Hospital, Drum Tower Hospital Group of Nanjing, Jiangsu, China; ^3^ Department of Obstetrics and Gynaecology, The Affiliated Suqian First People’s Hospital of Nanjing Medical University, Jiangsu, China

**Keywords:** sleep deprivation, memory impairment, cognitive function, lifestyle, physical therapy

## Abstract

Sleep’s crucial role in memory consolidation is well-established, with neuroimaging and sleep stage analysis revealing the intricate processes involved. Sleep deprivation significantly impairs memory performance and the ability to form new memories, highlighting the need for effective countermeasures. This article concludes that while sleep deprivation significantly impairs memory, the emerging insights into the gut-brain axis offer a promising frontier for developing novel interventions that can mitigate these effects. The review discusses various interventions, ranging from pharmaceutical compounds like donepezil, memantine, and tolcapone, to innovative physical therapy techniques such as transcranial magnetic stimulation (TMS), deep brain stimulation (DBS), and transcranial direct current stimulation (tDCS). Additionally, the emerging role of the gut-brain axis in sleep deprivation-induced memory impairment is examined, shedding light on the complex interplay between gut microbiota and cognitive functions. This comprehensive review explores the multifaceted relationship between sleep deprivation and memory impairment, delving into the neurobiological mechanisms underlying these processes and examining potential interventions.

## Introduction

Although our understanding of sleep’s functions is still limited, its role in memory consolidation is clear (1). Research demonstrates that both total sleep deprivation and partial sleep loss significantly impair memory tasks performance and the ability to form new memories (2, 3). Neuroimaging studies further reveal that sleep deprivation after learning crucially affects the long-term restructuring of memories in the brain (4). This review will examine the evidence of memory impairments induced by sleep deprivation and explore potential interventions to mitigate these effects. The inclusion of the gut-brain axis in the discussion of sleep deprivation and memory impairment advances the field by offering a novel perspective on how systemic health, particularly gut microbiota, can influence cognitive functions. This insight not only expands our understanding of the neurobiological mechanisms involved but also opens up potential new avenues for therapeutic interventions that could address both cognitive and systemic health issues.

The earliest studies highlighting the positive impact of sleep on memory date back to the 1920s (5). Researchers discovered that participants recalled syllables better if they slept after learning them, compared to staying awake. Presently, the concept of active systems consolidation during sleep lists among the most widely acknowledged theory explaining sleep-dependent memory consolidation (6). During learning or encoding new experiences, different aspects are processed and stored in various brain regions. The hippocampus plays a crucial role in integrating these components into a single episodic memory (7, 8). Sleep triggers the repeated reactivation of hippocampal neurons, which strengthens the memory representations in the neocortex through synaptic consolidation processes, leading to long-term memory formation (9).

Neuronal replay, essential for active systems consolidation, is predominantly observed during slow-wave sleep (SWS) and sometimes in wakefulness (10–12). This replay facilitates the formation of stable representations in extra-hippocampal networks, underlining the importance of sleep for memory consolidation. Advances in neuroimaging technologies like fMRI and EEG have provided more indirect evidence of memory replay during sleep (13, 14). Intracranial recordings in epilepsy patients have shown that gamma-band patterns, specific to the encoding of pictures, are crucial (15). Neuronal replay not only aids in system consolidation (the redistribution of hippocampus-dependent memories to neocortical sites for long-term storage) but may also contribute to synaptic consolidation (the enduring synaptic changes that stabilize memories).

**Figure 1:**
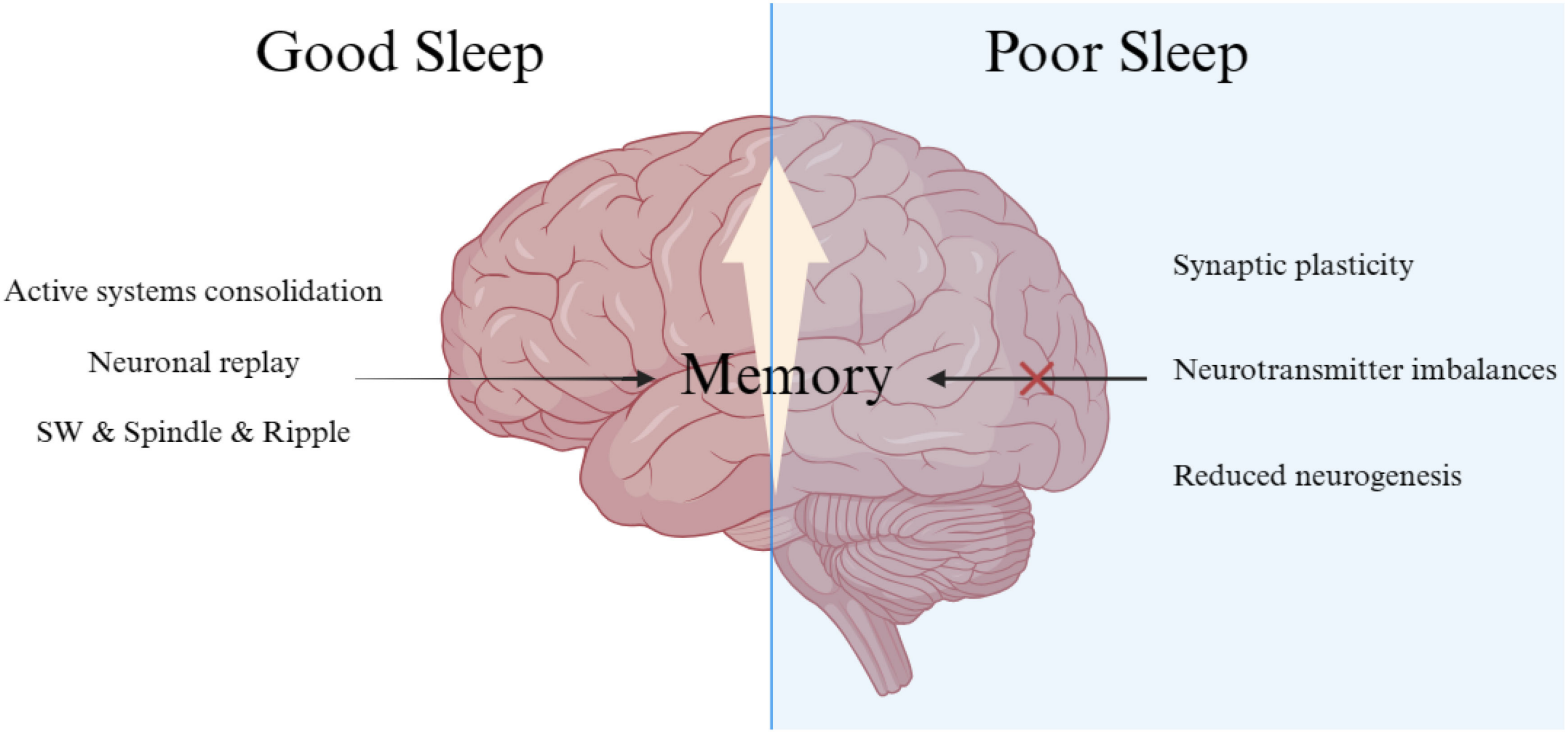
Neurobiological impact of sleep deprivation on memory.

Different sleep stages and specific wave patterns require special consideration. As mentioned, neuronal reactivation of spatio-temporal patterns during encoding primarily occurs in SWS. Neocortical slow oscillations (<1 Hz), thalamo-cortical spindles, and hippocampal sharp-wave ripples play a pivotal role in memory consolidation during SWS (9). During Rapid-eye-movement (REM) sleep, synaptic consolidation in the cortex may benefit from increased plasticity-related immediate-early gene activity. This stage is characterized by high-frequency brain waves, significant cholinergic, and theta activity. The slow oscillations’ depolarizing up-states initiate the formation of spindles and ripples, crucial for reactivating hippocampal memories and facilitating their efficient transfer to the neocortex (16). The alternating up- and down-states in neuronal activity induced by the neocortex’s slow oscillations create a higher-level temporal framework essential for redistributing memories for long-term storage (17, 18). Thalamo-cortical spindles prime cortical networks for long-term memory storage, with repeated spindle-associated discharges inducing long-term potentiation (19). This often occurs at synapses strengthened during memory encoding. Additionally, hippocampal sharp wave-ripples linked to sleep are associated with the reactivation of neuronal groups active during previous wakefulness (20, 21) (**Figure 1**).

## Lifestyle factors, sleep health, and cognitive function

Recent studies have highlighted the intricate relationship between lifestyle factors, sleep health, and cognitive function. A large cross-sectional study in China explored the impact of various healthy lifestyle habits, including diet, exercise, and weight management, on sleep quality and duration, revealing that individuals adhering to healthier lifestyles experienced better sleep outcomes, including a lower incidence of insomnia and obstructive sleep apnea (22). In contrast, a meta-analysis investigating the health implications of habitual daytime napping found that longer naps (30 minutes or more) were associated with an increased risk of adverse health outcomes, such as cardiovascular disease and metabolic disorders (23). Interestingly, shorter naps did not pose significant risks, suggesting that while a healthy lifestyle can improve sleep health, the benefits of daytime napping are nuanced and dependent on nap duration. These findings underscore the importance of balanced lifestyle choices in promoting overall health and cognitive well-being.

The intricate relationship between sleep and memory has been a subject of extensive research, revealing that sleep plays a crucial role in both consolidating memories and preparing the brain for new memory formation. Sleep deprivation, therefore, has significant adverse effects on memory, which is a growing concern in today’s society where sleep curtailment is increasingly common due to various lifestyle factors (24). Studies have consistently shown that sleep deprivation impairs memory performance (25, 26). For example, participants who are deprived of sleep after learning new information tend to perform worse in memory tests. This finding supports theories that emphasize the importance of sleep in memory consolidation – the process by which short-term memories are converted into long-term ones. Additionally, sleep-deprived individuals have a reduced capacity to encode new memories when compared to well-rested controls, indicating that sleep is essential not just for consolidating existing memories but also for forming new ones.

## Potential specific interventions

In our modern society, sleep deprivation is increasingly widespread, driven by factors ranging from the pervasive use of smartphones and other electronic devices to academic pressures and stress, particularly among adolescents (27–29). This widespread reduction in sleep quality and quantity necessitates finding effective methods to counteract the negative impact of sleep deprivation on memory. Among the countermeasures to combat the effects of sleep deprivation, substances like caffeine, amphetamines, and modafinil are known for their ability to enhance alertness and vigilance (30). However, more specific interventions have been explored in recent research. For instance, donepezil, a cholinesterase inhibitor typically used in the treatment of Alzheimer’s disease, has been investigated for its potential to mitigate memory deficits associated with sleep deprivation (31). Studies have found that while donepezil does not offer significant benefits to well-rested individuals, it can improve performance in tasks related to memory among those who are sleep-deprived. This improvement is particularly noticeable in individuals who experience a significant decline in performance due to lack of sleep. These findings are supported by neuroimaging studies showing changes in brain activity related to the task in areas affected by donepezil, suggesting that it may enhance delayed recognition in sleep-deprived individuals by improving both attention and memory encoding.

Another compound, tolcapone – a selective inhibitor of catechol-O-methyltransferase (COMT) – improves cognition and cortical information processing in rested individuals (32). However, its effectiveness against sleep deprivation-induced memory impairment appears to be influenced by the individual’s COMT genotype, highlighting the complexity of neurochemical interactions in sleep and memory processes (33). The use of donepezil and memantine, both approved for treating Alzheimer’s disease, has also shown promise in preventing memory impairment induced by sleep deprivation (34). This is significant as it not only supports the potential use of these drugs in a wider context but also underscores the similarity in the underlying neurobiological mechanisms involved in sleep deprivation and neurodegenerative diseases like Alzheimer’s.

## Gut-brain axis

Recent research has turned its focus to the gut-brain axis, particularly the role of gut microbiota in sleep deprivation-induced memory impairment. Insomnia and other sleep disturbances can lead to significant changes in the gut microbiome, which in turn can impact cognitive functions. Studies have demonstrated that sleep-deprived mice exhibit disturbed intestinal microflora and intestinal barrier dysfunction (35). Interestingly, healthy mice that received gut microbiota transplants from insomnia-affected donors exhibited cognitive impairments, suggesting a direct link between gut health and brain function (36). Researchers have found that the administration of melatonin, a hormone known to regulate sleep, can ameliorate cognitive impairments induced by sleep deprivation. This effect is mediated through alterations in gut microbiota and their metabolites, which in turn modulate inflammatory responses and neuronal apoptosis in the hippocampus, a key brain region involved in memory (37). The gut-brain axis operates through several mechanistic pathways, including the modulation of the vagus nerve by gut microbiota, which in turn influences brain function. Recent studies suggest that disruptions in gut microbiota caused by sleep deprivation can lead to systemic inflammation, which exacerbates cognitive decline. Understanding these pathways is crucial for developing interventions that not only target cognitive symptoms but also address underlying systemic issues, potentially leading to more effective and holistic treatments.

## Physical therapy

Exploring the enhancement of memory following sleep deprivation, physical therapy emerges as a promising method (38). This approach leverages physical factors—namely sound, light, electricity, and mechanical force—to modulate brain functions. Various forms of physical therapy, including transcranial magnetic stimulation (TMS), deep brain stimulation (DBS), and transcranial direct current stimulation (tDCS), have undergone investigation for their potential to mitigate memory impairments induced by sleep deprivation (39). Recent studies have demonstrated the efficacy of 1 Hz repetitive TMS (rTMS) in ameliorating spatial learning and memory deficits in sleep-deprived rats, attributed to the enhancement of synaptic structure and quantity in the hippocampus (39). Additionally, tDCS has shown promise in improving cognitive function following sleep deprivation without adversely affecting subsequent recovery sleep or post-recovery cognitive performance (40).

## Conclusion

These findings highlight the complexity of the mechanisms underlying sleep deprivation-induced memory impairment and point towards a multifaceted approach to mitigating its effects. The exploration of chemical interventions like donepezil, memantine, compounds targeting the gut-brain axis, and physical therapy like rTMS and tDCS opens new avenues for research and potential treatments. However, these interventions should be considered alongside lifestyle changes that promote healthy sleep patterns, as the benefits of good sleep hygiene cannot be overstated. As research continues to unravel the intricate connections between sleep, the gut microbiome, and memory, a more holistic understanding of these processes will emerge, paving the way for more effective treatments and preventative strategies.
